# Four years of experience with carbapenem-resistant Gram-negative bacteria in two tertiary care hospitals in Crete, Greece

**DOI:** 10.1186/2047-2994-4-S1-P130

**Published:** 2015-06-16

**Authors:** C Tsioutis, S Karageorgos, S Stratakou, E Bolikas, E Astrinaki, A Messaritaki, M Adami, A Georgiladakis, A Kassimati, A Gikas

**Affiliations:** 1 Infection Control Unit, University Hospital of Heraklion, Heraklion, Crete, Greece; 2 Internal Medicine / Infectious Diseases, University Hospital of Heraklion, Heraklion, Crete, Greece; 3 Infection Control Unit, Venizelion General Hospital, Heraklion, Crete, Greece; 4 Department of Microbiology, University Hospital of Heraklion, Heraklion, Crete, Greece; 5 Department of Microbiology, Venizelion General Hospital, Heraklion, Crete, Greece

## Introduction

Carbapenem-resistant gram-negative bacteria (CRGNB, *Acinetobacter baumannii*, *Klebsiella pneumoniae*, *Pseudomonas aeruginosa*)) are important nosocomial pathogens in Greece.

## Objectives

To describe the epidemiology of CRGNB in two tertiary care hospitals in Crete, Greece.

## Methods

Analysis of infection control records from June 2011 to December 2014 of 450-bed Venizelion Hospital and 750-bed University Hospital, both being referral centres for Southern Greece. Consecutive patients with CRGNB isolation (only first CRGNB per patient) were recorded. Data presented as no.(%) or mean±SD.

## Results

A total of 1537 cases with CRGNB were detected: 582 *A. baumannii*, 510 *K. pneumoniae* and 445 *P. aeruginosa*. Mean patient age was 63.6±18.7 years. The greatest burden was in ICU (41.4%) and medical wards (36.7%). Respiratory specimens constituted the most frequent source of *A. baumannii* (56.3%) and *P. aeruginosa* (43.6%); urine samples were the most frequent source of *K. pneumoniae* (28.8%). In-hospital mortality (37.7%) was similar among the 3 pathogen groups; however, ICU patients with CRGNB had higher mortality rates compared to other departments (53.5% vs 26.6%, p<0.001). Similarly, ICU patients had longer hospital stay after CRGNB isolation compared to other departments (median 21 vs 8 days, p<0.001).

## Conclusion

This study shows that the burden of CRGNB is much greater in ICUs, accounting for significant morbidity and mortality. Therefore, in Greek hospitals where CRGNB infections are a major problem, infection control measures should mainly focus in critical care departments.

## Disclosure of interest

None declared.

